# Cryoablation of Primary Breast Cancer in Patients with Metastatic Disease: Considerations Arising from a Single-Centre Data Analysis

**DOI:** 10.1155/2017/3839012

**Published:** 2017-10-19

**Authors:** Claudio Pusceddu, Luca Melis, Nicola Ballicu, Paolo Meloni, Valeria Sanna, Alberto Porcu, Alessandro Fancellu

**Affiliations:** ^1^Division of Interventional Radiology, Oncological Hospital “A. Businco”, Cagliari, Italy; ^2^Division of Nuclear Medicine, Oncological Hospital “A. Businco”, Cagliari, Italy; ^3^Unit of Medical Oncology, Hospital “SS. Annunziata”, Sassari, Italy; ^4^Unit of General Surgery 2, Clinica Chirurgica, Department of Clinical and Experimental Medicine, University of Sassari, Sassari, Italy

## Abstract

**Background:**

Patients presenting with stage IV breast cancer might benefit by removal of the primary tumor. We report our experience with CT-guided cryoablation of the primary tumor, with the aim of evaluating its role in this subgroup of patients.

**Patients and Methods:**

Data of 35 patients with mean age of 58 years with breast cancer at stage IV submitted to CT-guided cryoablation of the primary tumor between 2010 and 2016 were prospectively evaluated. All patients, except three, were preoperatively and postoperatively evaluated with breast MRI to assess the extent of tumor necrosis. Retreatment was performed in case of incomplete ablation.

**Results:**

Mean tumor size was 3.02 ± 1.4 cm. Six patients had multicentric disease. Complete tumor necrosis was 85.7% and 100% at 2-month and 6-month follow-up, respectively, as 5 patients with tumors > 3 cm required a redo cryoablation. No patient developed major complications. Minor side effects occurred in 30 patients (82%). All patients were discharged the same day of the procedure. During a mean follow-up of 46 months (range 3–84), 7 patients (20%) experienced local recurrences that were treated with redo cryoablation, and 7 (20%) died for disease progression.

**Conclusions:**

Our results suggest that cryoablation of the primary tumor is safe and effective in the treatment of patients presenting with stage IV breast cancer.

## 1. Introduction

The widespread use of screening mammography has led to a significant increase in detection of early breast cancer [1, 2]. However, patients presenting with metastatic disease at the time of the primary diagnosis account for about 5–10% of patients with breast cancer [3]. They are commonly considered to have a dismal prognosis, with a 5-year overall survival of less than 30% [4]. Removal of the primary tumor in that subgroup of patients has been usually reserved to cases in which palliation was deemed necessary due to local complications, while it has been historically avoided in patients with asymptomatic breast cancer. The rationale behind this approach is that it does not seem useful to locally treat the primary tumor in presence of disseminated disease. However, some authors have recently reported that resection of the breast tumor may result in survival benefits in patients presenting with distant metastases at the time of diagnosis (stage IV according to UICC/AJCC staging system) [3, 5, 6].

In recent years, applications of image-guided percutaneous ablation techniques are on the rise in patients with breast cancer. In this regard, different ablative methods have been currently used, such as cryoablation, laser irradiation, microwave irradiation, radiofrequency ablation, high-intensity focused ultrasound ablation, and irreversible electroporation [7–9]. Among them, cryoablation has been considered one of the most efficacious techniques for obtaining tumor necrosis [8, 10]. Essentially, the efficacy of cryoablation is due to cytotoxic effects of cold that produces destruction of cellular ultrastructure at temperatures lower than −40°C [10–12]. Recent reports highlighted a possible effect of thermal ablation on immunomodulation other than simple tumor destruction [13]. Cryoablation of breast cancer presents some advantages for less-invasive treatment of breast cancer when compared with surgical lumpectomy, although several aspects still remain to be elucidated. To date, studies on this topic have been focused on the use of cryoablation as alternative to surgery in treatment of selected patients with small operable breast cancer. However, studies focusing on the role of cryoablation in patients with breast cancer at stage IV are scarce in the current literature.

In a previous paper, we pointed out that cryoablation of primary breast cancer in patients with bone metastases is a well-tolerated, feasible, and effective treatment option [10]. The aim of the current study is to evaluate the safety and efficacy of cryoablation in achieving tumor ablation in patients with stage IV breast cancer.

## 2. Material and Methods

### 2.1. Study Population

In 2010 we started a program aimed at treating with cryoablation patients presenting with metastatic breast cancer. Data from every patient with metastatic breast cancer who underwent CT-guided cryoablation of the primary tumor between 2010 and 2016 has been collected in an institutional review board-approved database, in order to conduct a prospective study. For the present study, inclusion criteria were as follows: (a) patients with invasive breast cancer presenting at stage IV according to UICC/AJCC staging system, (b) primary breast tumor without skin infiltration, in order to avoid skin burn during the cryoablation procedure, and (c) life expectancy greater than 12 months. Criteria for exclusion were platelet count <50/mm^3^ or unmanageable coagulation disorders.

Decision on cryoablation treatment was taken after multidisciplinary team meeting involving breast surgeons, medical oncologists, radiotherapists, and interventional radiologists. Informed consent was obtained from all patients.

### 2.2. Preoperative Work-Up

Two weeks before the cryoablation procedure the patients underwent preoperative laboratory examinations and imaging work-up including mammography and both nonenhanced and contrast-enhanced MRI imaging. The latter was considered the preferred imaging modality to assess the tumor characteristics such as size and contrast enhancement. MR images were acquired with the patients in prone position in a 1.5-Tesla system (MAGNETOM Avanto; Siemens, Germany). Radiofrequency signal reception was obtained by means of a dedicated sensitivity-encoding breast coil. Six dynamic acquisitions of T1-weighted three-dimensional fast low-angle shot sequences were obtained for each MRI session. After administration of 0.1 mL/kg gadobutrol (Gadovist; Schering AG, Germany), intensity/time enhancement curves were obtained by drawing a region of interest (ROI) around the areas of the breast lesions that had the greatest degree of enhancement. All CT images were acquired with a Somatom Sensation CT scanner (Siemens) with 3-mm collimation and 80–140 mA. Before cryoablation, three-dimensional tumor measurements were done on CT images. The density of each tumor was measured as CT attenuation coefficient in Hounsfield units (HU) by drawing a ROI around the tumor.

### 2.3. Cryoablation Procedure

All cryoablation sessions were performed under CT guidance by a single board-certified interventional radiologist as described in our previous paper [10]. Briefly, percutaneous cryoablation was carried out using an argon-based cryoablation unit (SeedNet; Galil Medical, Yokneam, Israel). Both preoperative CT and MRI images were compared in order to determine the correct positioning of the probes for tumor ablation. In particular, attention was given in evaluating the different positioning and compression effects determined by the supine versus prone position on CT and MR images, in order to adjust the probe(s) position for minimal discrepancies in the size and location of the target lesions. All the procedures were carried out under conscious sedation, using intravenous bolus of fentanyl citrate 50 *μ*g, and local anesthesia. Vital signs were continuously monitored throughout the procedure. Local anesthesia was given by injection of 2–5 mL of 2% lidocaine proximal to the tumor lesion(s) and along the course of the cryoprobes. One or more cryoprobes (1.47-mm diameter 17-gauge ISOTHERM IceRod, IceRod Plus, and IceSphere needles; Galil Medical) were inserted into the targeted tumor using CT guidance, through a small skin incision of 1-2 mm. To avoid cryoinduced skin injury and facilitate the ablation of breast tissue at least 1 cm beyond all apparent tumor margins, warming bags were placed on the breast skin. In 6 patients (17.1%), in whom the breast tumor was multicentric, multiple cryoprobes were inserted approximately 1.5 cm apart and <1 cm from all tumor margins according to established guidelines, in order to generate cytotoxic isotherms in almost any tissue [10]. Due the relatively low heat load of breast parenchyma compared with internal organs, it was estimated that 1 cm of visible ice beyond all the tumor margins would be necessary to generate cytotoxic temperatures (e.g., −40°C) throughout the breast tumor [14]. In two patients with large axillary lymph node involvement and in one patient with painful sternal bone metastases, the breast primary tumor and the metastases were treated in the same cryoablation session. Each cryoablation treatment session consisted in two cycles of 8 minutes of duration, followed by a 4-minute active thawing phase and a 4-minute passive thawing phase. The latter is considered useful to maximize cell death [15]. CT images were acquired at the end of each phase of freezing cycle, in order to verify the formation of a homogeneous area of low density, owing to the iceball, which encompassed the tumor all around. The probes were removed after the second phase of thawing, without the need for skin suture. CT images were acquired immediately after the end of the cryoablation procedures to detect early complications. After the procedure, the patients were transferred to the recovery room and discovered after 6–10 hours after the ablation, with a prescription for an oral wide-spectrum antibiotic therapy for 72 hours. The clinical outcome was evaluated at the end of the cryoablation procedure and 1 month after, based on the presence or absence of four main parameters: appreciable nodular thickening on palpation, skin rash, bruising, and hyperpigmentation. The absence of such signs or symptoms was interpreted as an optimal clinical result.

### 2.4. Imaging Follow-Up

**Figure 1:**
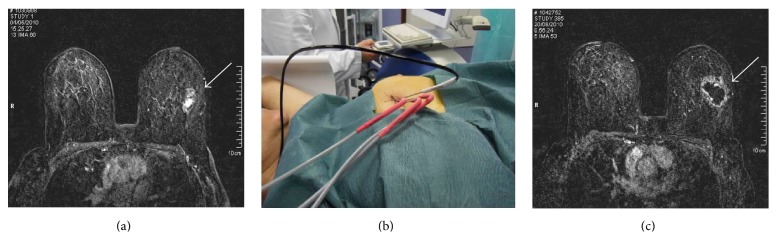
A 50-year-old woman was diagnosed as having invasive ductal carcinoma and distant metastases. Axial contrast-enhanced T1-weighted fat suppression MR image showed the primary breast cancer located in the external quadrant of the left breast measuring 27 mm in its major diameter (arrow) (a). She was submitted to CT-guided cryoablation of the breast tumor under local anesthesia and conscious sedation. We used three cryoprobes along with one thermocouple for temperature monitoring (b). Axial contrast-enhanced T1-weighted fat suppression image 2 months after the procedure showed complete ablation with a large nonenhanced area, due to tissue necrosis, surrounded by a ring of enhanced tissue compatible with granulation tissue in the proliferative phase (arrow) (c).

Results of the ablative procedure outcomes were radiologically evaluated by breast MR imaging or CT scan in claustrophobic patients, at intervals of 2, 6, and 12 months after cryoablation treatment and yearly thereafter. Acquisition protocols for MRI/CT scans were the same as those used for the assessment of the characteristics of breast tumor lesions before the cryoablation procedure. At 2- and 6-month imaging follow-up, the complete loss of contrast enhancement on either MRI or CT scans was considered a complete response to ablative therapy (Figure 1). At 12-month follow-up, any changes observed on either breast MR or CT scans in the contrast enhancement or any increases in the size of the treated lesions were considered as tumor recurrences or disease progression. The decision regarding timing and mode of the radiologic outcome assessment was principally based on the evidence that early postoperative MR imaging or CT in the first 30 days after the procedure may be hampered by a rim of strong enhancement of treated lesions. This finding is linked to the inflammatory injury induced by cryoablation itself [15].

### 2.5. Outcomes of Interest

For the purposes of this study, the following data were extrapolated for each patient: age, tumor size, tumor distribution (unicentric or multicentric), histotype, site of distant metastases, findings of the preoperative breast imaging examinations (tumor enhancement at MRI, distance from the skin and chest wall), number of probes used for cryoablation, number of sessions, complications, hospital stay, rates of complete tumor necrosis at 2 and 6 months, and rates of local recurrence and survival after treatment. Fisher exact test was used to evaluate rates of complete tumor ablation according to preoperative tumor size. Time to local tumor progression was estimated by Kaplan–Meier analysis. Analysis was conducted using IBM SPSS Statistics version 20 (IBM Corporation, 2011).

## 3. Results

**Figure 2:**
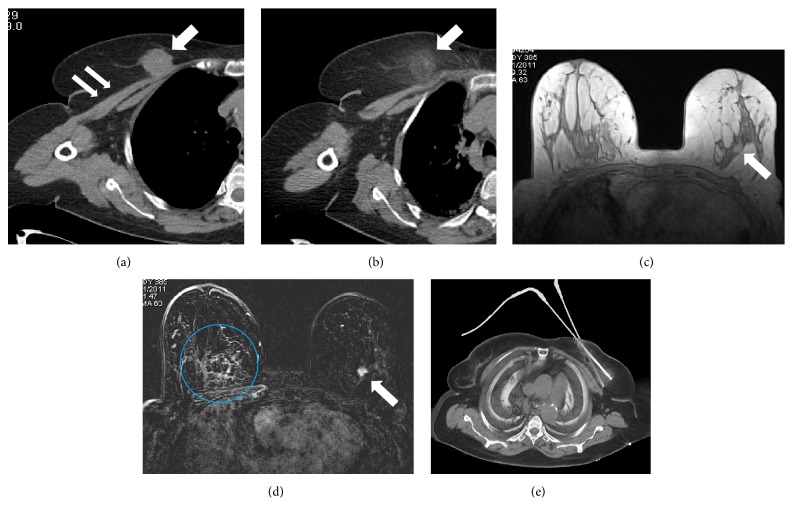
CT scan of an 81-year-old woman using the mediastinal window setting showed a primary breast cancer of the right breast (arrow) infiltrating the major pectoralis muscle (double arrow) (a). The patient received cryoablation of the primary tumor. CT scan obtained at the end of the cryoablation procedure showed the presence of a homogeneous area of low density because of the iceball (arrow), which encompassed the tumor (b). The same patient developed a contralateral breast cancer after 13 months. T2-weighted MR image showed a breast tumor in the left breast (arrow) (c). Contrast-enhanced T1-weighted fat suppression MR image showed the complete ablation of the cancer of the right breast and the contralateral tumor (circle and arrow, resp.) (d). CT-guided cryoablation of the left breast cancer using two cryoprobes (e).

Thirty-five patients with stage IV breast cancer underwent CT-guided cryoablation of the primary tumor in the considered period. Preoperative work-up included breast MR imaging in 32 patients (91.4%) and CT scan of the breast in the 3 claustrophobic patients (8.6%). Mean patients' age was 58 ± 12 years. Breast cancer was multicentric in 17.1% of patients. Mean tumor size (evaluated at preoperative MRI) was 3 ± 1.4 cm. Invasive ductal carcinoma was the predominant histotype (91.4%). Site of distant metastases was skeleton in the majority of cases (85.7%); only one patient (2.8%) had multiple-site metastases (skeleton and liver). The characteristics of the study population are resumed in Table 1. All cryoablation cases were well tolerated and patients were discharged 6–10 hours after treatment in all cases. We did not observe any major procedure-related complications, such as hematoma formation, breast infection, and skin burn. Minor side effects including ecchymosis, oedema, and skin pigmentation occurred in 30 patients (82%). These signs were transient and resolved spontaneously within 20 days after the procedure. Other than the initial 35 cryoablation sessions performed as upfront treatment of primary breast cancer, cryoablation was repeated for incomplete necrosis after two months in 5 cases (14.3%) and for local recurrence in 7 patients (20%); in one patient (2.8%) cryoablation was carried out for occurrence of a new cancer in the contralateral breast (Figure 2). It accounts for a total of 48 cryoablation procedures in 35 patients.

**Figure 3:**
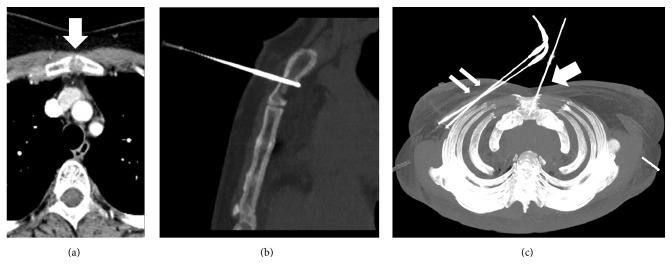
A 70-year-old woman having a ductal breast carcinoma of the right breast and a painful osteolytic metastasis of the sternal bone (arrow) (a). A simultaneous cryoablation of the primary breast tumor (double arrow) and of the bone metastasis was performed (arrow) (b, c).

**Figure 4:**
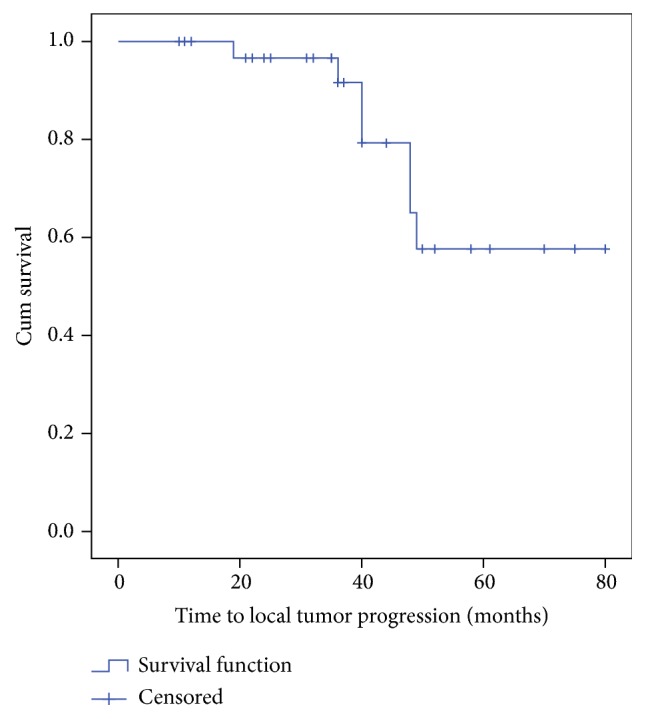
Kaplan–Meier curve for time to local recurrence in the 35 patients of the study cohort.

Data relative to the cryoablation procedures in the study population are resumed in Table 2. All sessions of CT-guided cryoablation were successfully completed. In fact, CT scans obtained at the end of the ablation procedures showed an extent of the iceball at least 1 cm beyond the limits of the tumor margins, and this was considered a safety margin. After primary ablation we observed complete tumor necrosis in 30 patients (85.7%), whereas, in 5 patients (14.3%) with tumors > 3 cm in diameter, a second cryoablation was needed to ensure complete ablation. Thus, rates of complete tumor necrosis after primary treatment became significantly higher in patients with tumor size < 3 cm (*p* = 0.02). In those patients with incomplete tumor ablation after the first session, residual disease was noted at MRI follow-up, possibly as a result of an incomplete overlap of the iceball. After secondary treatment, complete tumor ablation was observed in 100% of cases. The complete response to treatment was demonstrated by MRI imaging at 6-year follow-up MRI. In two patients (5.7%) who presented large and symptomatic axillary lymph node metastasis simultaneous cryoablation was performed. Another patient presenting with breast cancer and painful sternal bone metastasis was also treated with simultaneous cryoablation of both breast and bone (Figure 3). After a median follow-up of 46 months (3–84), seven patients (20%) experienced local recurrence and were retreated with cryoablation, and seven patients (20%) died because of tumor progression. Mean time to local recurrence was 64.0 ± 4.7 months (confidence interval 54.7–73.2) (Figure 4).

## 4. Discussion

The need for local treatment of the primary tumor in addition to systemic therapy for patients presenting at stage IV breast cancer remains an open issue [16]. While lumpectomy has been considered mandatory in presence of local complications, such as bleeding, infection, or ulceration, asymptomatic breast tumors have been usually left intact [17]. In fact, resection of the primary tumor may expose patients to possible surgical complications and delay the start of systemic treatments without any demonstrated benefit in terms of survival. On the other hand, distant metastases may be supported by different neoplastic cell lines; thus resection of the primary breast cancer may result in removal of a potential source of tumor stem cells with metastatic potential, from a theoretical point of view [17]. Some retrospective studies reported advantages in overall survival in patients undergoing resection of the primary tumor when compared with those in whom the breast primary tumor was not removed [6, 18, 19]. Conversely, prospective trials having as main outcomes the survival benefits after resection of the primary breast cancer in patients with metastatic disease, have reached contradictory results [5, 17]. In a recent meta-analysis evaluating almost 30,000 stage IV breast cancer patients from 16 comparative studies, Headon and coworkers reported that surgery is beneficial in reducing the risk of mortality by 37% [5]. However, robust evidence on this issue is expected from the ongoing trials.

In the present scenario, the use of ablative methods for treatment of the primary breast tumor seems justified, because several percutaneous ablative methods have been proposed in place of surgical resection in recent years [7–9, 20, 21].

In the experience reported in this paper, we preferred the use of cryoablation because it presents many advantages with respect to other ablative techniques; in fact, cryoablation bears the possibility of treating larger lesions by using more than one cryoprobe, causes less pain due to the intrinsic pain control of the tissue cooling, and can be performed under local anesthesia and conscious sedation. Of note, all patients in our report were discharged the same day following the procedure, and none of them developed major complications. Recently, the use of cryoablation in breast cancer has been proposed as alternative to surgical resection for small tumors, and also in patients who were unsuitable for surgical treatment or who refused surgical resection, with encouraging results [22–24]. In the ACOSOG Z1072 phase II trial, with 99 patients with unifocal invasive ductal carcinoma ≤ 2 cm and intraductal component < 25%, the successful ablation of breast cancer was 75.9% and raised to 92% if multifocal disease outside of the targeted cryoablation tumor zone was not considered as an ablation failure [24].

However, only few studies have investigated the use of cryoablation as ablative method in patients at stage IV. One of them was our previous study, based on data from 17 patients with skeletal metastases who received cryoablation of the primary tumor, where we observed a complete regression of the breast tumor in 88% of patients two months after treatment [10]. In the present study, we presented also data referring to patients with metastatic disease in different places other than skeleton, such as liver and lung, although an expected life expectancy of at least 12 months was considered an inclusion criterion. With respect to our previous experience, we have widened indications to patients with invasive lobular carcinoma and to those with tumors located <1 cm from the skin, on condition that skin infiltration was absent. Another study described the feasibility and efficacy of breast cryoablation in only six patients with stage IV breast cancer [14].

The rates of complete ablation vary widely in the literature [8, 22]. In recent meta-analysis from Mauri and coll. the efficacy of cryoablation (defined as the rate of lesions completely ablated) ranged from 51 to 90% [9]. In the present report, we obtained a rate of total necrosis of 85.7% after 2 months and of 100% after 6 months, being the initial incomplete necrosis observed in patients with large tumors (>3 cm).

As for follow-up imaging after cryoablation, we preferred the use of MRI, because several studies demonstrated that MRI has high accuracy in evaluating the extension of breast cancer [21, 22, 25, 26].

Although ultrasound guidance is commonly used for monitoring the freezing process during cryoablation, it has some limitations. Indeed, accurate monitoring of the iceball formation is hampered by the acoustic shadowing effect [10]. CT guidance allows monitoring of the entire iceball formation during the freezing phases. Although we performed all procedures under CT guidance, it should be recognized that ultrasound guidance may be helpful for needle insertion. A combined approach with needle insertion under ultrasound guidance and following ablation monitoring with ultrasound and CT may be useful, similar to what it can be done during image-guided ablative procedures in abdominal organs. MRI guidance for cryoablation is a useful method but it is restricted to few centres due to its high cost.

Cryoablation is a versatile method for treatment of breast neoplastic lesions, due to its considerable cryodestructive potential of the multiprobe freeze approach. In fact, 17,4% of patients in our cohort received treatment for multicentric breast cancer; 5 patients had redo cryotherapy for incomplete ablation and 7 for local recurrence. Moreover, cryoablation in stage IV breast cancer may permit the simultaneous treatment of primary cancer and also close symptomatic metastases. In our cohort of patients we cryoablated two large axillary lymph node metastases and one sternal bone metastasis without complications.

Another interesting aspect of cryotherapy is its possible antitumoral immunity effects. Studies have suggested that tumor-specific immunoresponses stimulated by the cryoablated tissue may have a role in controlling the development of metastases distant from the primary tumor site [13, 27].

In our study, the patients did not undergo surgical resection of the breast tumor after cryoablation; thus a comparison between these two approaches cannot be done. However, we can speculate that cryoablation has several potential advantages when compared to surgical resection in patients at stage IV. First, it can be carried out under local anesthesia and conscious sedation, without the need of night hospital stay. Moreover, cryoablation does not require surgical incisions, avoids the risk of surgical complications, does not need to interrupt or delay systemic treatment, which remain the mainstay for management of those patients, and it has higher cost-effectiveness. In comparison with surgery, cryoablation might require more than one treatment session in order to achieve complete tumor ablation; however this should not be considered as a disadvantage because complete tumor destruction can be obtained with a repeated minimally invasive procedure.

Our study present some limitations, the main being retrospective data analysis and small sample size. However, it should be taken into account that patients presenting at stage IV only account for a minority of cases diagnosed with breast cancer and also that this subgroup of patients traditionally has not been managed with either resection or ablation of the primary tumor. In this scenario, our paper represents the study with the higher number of patients regarding the use of cryoablation in stage IV breast cancer. Well designed, prospective studies comparing cryoablation and surgical resection are expected in the near future, for better understanding the role of this approach in this subgroup of patients and to evaluate possible survival benefits.

## 5. Conclusions

In summary, our results suggest that cryoablation of the primary tumor is safe and effective in the treatment of patients presenting with stage IV breast cancer.

## Figures and Tables

**Table 1:** Characteristics of the study population.

Characteristic	Number (%)
Age (years) *mean, SD, range*	58 ± 12 (37–81)
Affected breast	
Left	15 (42.85)
Right	20 (57.15)
Tumor distribution	
Upper external quadrant	13 (37.15)
Lower external quadrant	6 (17.14)
Upper internal quadrant	6 (17.14)
Lower internal quadrant	4 (11.43)
Multicentric	6 (17.14)
Histotype of breast carcinoma	
Invasive ductal	32 (91.4)
Invasive lobular	3 (8.6)
Tumour size (cm) *mean, SD, range*	3.02 ± 1.40 (1.3–6.7)
Site of distant metastases	
Skeleton	30 (85.71)
Liver	1 (2.86)
Lungs	3 (8.57)
Skeleton + liver	1 (2.86)

**Table 2:** Data relative to the cryoablation procedures in the study population.

	Number (%)
Total number of treatments performed	48 (100)
Upfront cryoablation treatment	35 (72.9)
Redo cryoablation for incomplete necrosis	5 (10.4)
Redo cryoablation for local recurrence	7 (14.6)
Cryoablation for new contralateral breast cancer	1 (2.1)
Types of probe used for cryoablations	
IceRod	48 (50.0)
IceRod Plus	28 (29.2)
IceSphere	20 (20.8)
Number of cryoprobes used per treatment	
1 cryoprobe	9 (18.7)
2 cryoprobes	30 (62.6)
3 cryoprobes	9 (18.7)
Synchronous cryoablation of metastases	
Axillary lymph node	2 (4.2)
Sternal bone	1 (2.1)
Two-month follow-up MRI/CT scan	
Complete tumor necrosis	30 (85.7)
Incomplete tumor necrosis	5 (14.2)
Six-month follow-up MRI/CT scan	
Complete tumor necrosis	35 (100)
Incomplete tumor necrosis	0 (0)
Complications	
Major complications	0 (0)
Minor side effects	30 (82%)
